# Seasonal and annual changes in PAH concentrations in a remote site in the Pacific Ocean

**DOI:** 10.1038/s41598-019-47409-9

**Published:** 2019-08-29

**Authors:** Kaori Miura, Kojiro Shimada, Taichi Sugiyama, Kei Sato, Akinori Takami, Chak K. Chan, In Sun Kim, Yong Pyo Kim, Neng-Huei Lin, Shiro Hatakeyama

**Affiliations:** 1 Graduate School of Agriculture, Tokyo University of Agriculture and Technology, Fuchu, Tokyo Japan; 2 Global Innovation Research Organization, Tokyo University of Agriculture and Technology, Fuchu, Tokyo Japan; 3 Graduate School of Creative Science and Engineering, Waseda University, Tokyo, Japan; 4 Graduate School of Engineering, Kyoto University, Kyoto, Japan; 5 National Institute for Environmental Studies, Tsukuba, Ibaraki Japan; 6 School of Energy and Environment, City University of Hong Kong, Hong Kong, China; 7 Department of Environmental Science & Engineering, Ewha Womans University, Seoul, Republic of Korea; 8 Department of Chemical, Engineering & Materials Science, Ewha Womans University, Seoul, Republic of Korea; 9 Department of Atmospheric Science and Department of Chemistry, National Central University, Chung-Li, Taiwan; 10 Center for Environmental Science in Saitama, Kazo, Saitama Japan

**Keywords:** Atmospheric chemistry, Environmental monitoring

## Abstract

This paper reports the long term observation of particle-associated polycyclic aromatic hydrocarbons (PAHs) at Cape Hedo Atmosphere and Aerosol Monitoring Station, a remote site in the Western Pacific Ocean, from 2008 to 2015. This is the first long-term study that evaluated the contribution of long-range transport of PAHs in East Asia. No obvious trend (*P* > 0.05) was found in a particular season over the years. However, there are seasonal variations of PAH concentrations with higher in spring and winter. The higher PAH are attributed to air masses from the area including part of China. Source apportionment using three different approaches, i.e., PAH compositional pattern analysis, PAH diagnostic ratio analysis and positive matrix factorization modeling, showed the combined high contribution of biomass burning (18%, 14%) and coal combustion (33%, 24%) in spring and winter. In addition, the contribution of ship emissions (35%) was relatively high in spring, whereas that of vehicle emissions (36%) was relatively high in winter. The contribution of coal combustion to PAH has decreased throughout the years, likely due to changes in energy structure in China. The contribution of biomass burning to PAH has showed no trend, being stable, and that of vehicular emissions has increased.

## Introduction

Polycyclic aromatic hydrocarbons (PAHs) are organic compounds that originate mainly from incomplete combustion of organic materials. They are primarily associated with fine particles, and their sources in ambient aerosol are vehicle exhaust and combustion of biomass and fossil fuel^1^. PAHs have been extensively investigated because of their mutagenic and carcinogenic potential^2^. Benzo[a]pyrene (Bap), one of the most carcinogenic PAHs, has been classified as carcinogenic to humans (Group 1) by International Agency for Research on Cancer^2^. Therefore, understanding the amount of PAHs in the atmosphere is important not only for effectively controlling of PAHs but also for evaluating associated health risks. Efforts have been made to estimate PAHs emission in the United States and Europe as well as East, Southeast, and South Asia. In particular, PAHs emission in East, Southeast, and South Asia is higher than other countries. PAHs emission sources are coal combustion in East Asia and biomass burning in Southeast, and South Asia. Moreover, the relative contribution of PAH emissions from motor vehicles is increasing under economic transition^3,4^.

We have made observations of atmospheric aerosols from 2008 to 2015 at the Cape Hedo Atmosphere and Aerosol Measurement Station (CHAAMS) in Okinawa, Japan, a remote site between East Asian Continent and Japan used in the Atmospheric Brown Cloud (ABC) project of the United Nations Environment Programme (UNEP) for investigating the long term transboundary air pollution in East, Southeast, and South Asia^5,6^. Using a potential receptor influence function model, Lang *et al*. suggested that PAHs emitted from northeastern China were transported to CHAAMS by northwesterly wind that occurred most frequently during winter-spring months^7^. Between 2000 and 2010, the fine-mode aerosol optical depth over CHAAMS increased and showed a peak around 2005–2006 and subsequently decreased based on observations by the Moderate Resolution Imaging Spectroradiometer and simulations by a chemical transport model^8^. The long term trend of PAH concentration, however, has not yet been reported in the literature.

Lately, the consumption of coal in China has reduced^9^, so are the emissions of air pollutants such as EC derived from coal combustion^10^. An important source of PAHs is coal combustion. For example, PAHs are major pollutants in China^11,12^; its high PAH emissions are attributed to intensive coal combustion and coke production, especially in northern China, where burning coal for home heating is widespread^5,13^. However, PAHs concentration in China still is from ten to a hundred times higher than in other East Asia and South East Asia countries^14^. The ambient PAH concentrations in Japanese and Korean cities from 2002 to 2014 have decreased significantly because of decreasing automobile contribution^15,16^. Therefore, understanding the change of PAHs source in the atmosphere is also important to evaluate for the long term trends of PAHs concentrations. We applied three different source apportionment methods (PAH compositional pattern, PAH diagnostic ratio analysis and Positive Matrix Factorization model) to identify the sources of PAHs in this study.

The objectives of this study are to (1) determine the long term trends of the PAHs concentrations at CHAAMS, (2) identify the sources of the observed PAHs based on long term data sets and (3) evaluate the impacts of reduction in emissions in China in these observed trends.

## Results and Discussion

### Intensive samplings at CHAAMS (2008–2015) Annual changes

**Figure 1:**
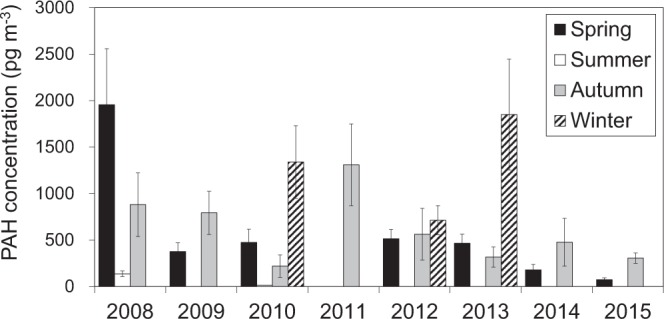
Average Σ15PAH concentration of each sampling period from 2008 to 2015.

Twenty-two intensive samplings were conducted from 2008 to 2015, and total of 172 samples were collected at CHAAMS. The average Σ15PAHs concentration of each sampling period is shown in Fig. 1. The sampling period, sample number, and TSP concentration of each sampling period are shown in Table S1. The average concentration was the highest in spring 2008 and lowest in summer 2010. The winter concentrations were highest in each year when winter samples were collected, and the average concentrations in winter in 2010, 2012 and 2013 were (13.3 ± 3.9) × 10^2^, (7.1 ± 1.6) × 10^2^, and (18.5 ± 6.0) × 10^2^ pg m^−3^, respectively. In contrast, summer concentrations were the lowest in each sampled year: the average concentrations in 2008 and 2010 were 138 ± 30 and 14.5 ± 1.1 pg m^−3^, respectively.

We tested for trends by applying the Mann-Kendall test, which is widely used to verify the significance of tendencies in datasets^17^. In any given season, no significant trend (*P* > 0.05) was found over the study period.

### Seasonal variations of PAH concentration and fraction of air mass origins

Seasonal variations of the average Σ15PAHs concentrations are shown in Fig. S1. The average concentration in spring (*N* = 78), summer (*N* = 13), autumn (*N* = 55), and winter (*N* = 26) were (6.1 ± 1.1) × 10^2^, (9.0 ± 2.5) × 10, (58.9 ± 9.1) × 10 and (12.9 ± 2.6) × 10^2^ pg m^−3^, respectively. The average concentration was the highest in winter and the lowest in summer (winter > spring ≈ autumn > summer). Note that the concentration in winter was about 14 times higher than that in summer. A similar seasonal trend was reported by Sato *et al*., who observed PAHs at CHAAMS from 2005 to 2008^18^.

There are two factors that could potentially influence seasonal variation of PAH concentration at CHAAMS: (1) affection of aerosol transportation by meteorological parameters and (2) origin of the air mass. In terms of meteorological parameters, PAHs concentrations depend on their gas/particle phase partitioning and photochemical aging. The lower molecular weight compounds such as three and four-ringed compounds (PHE, FLT, PYR etc.,) are prone to affected by gas/particle partitioning due to temperature effect. In this study, three and four-ringed PAHs concentrations were higher in winter than in spring (see following section).

**Figure 2:**
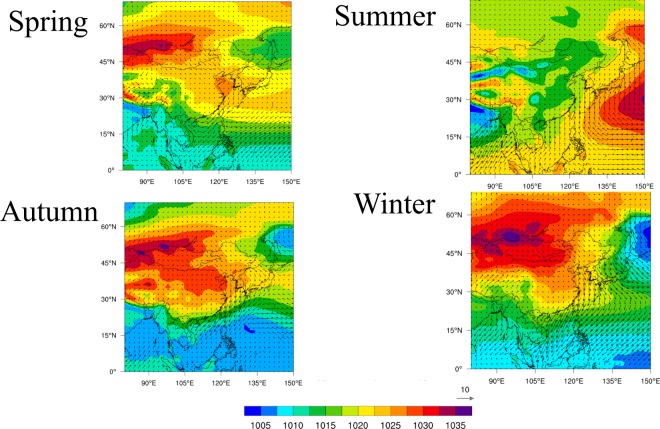
Seasonal mean sea level pressure (hPa) over East Asia and mean horizontal winds (u, v).

The flow of air masses is governed by the wind field, created by pressure gradients as shown in Fig. 2 in which the seasonal mean wind fields from 2008 to 2015 are shown. The high pressure was located over northwestern China with low pressure over the western Pacific Ocean during winter, spring and fall. On the other hand, the pressure pattern in summer was located over the western Pacific Ocean. Consequently, the air mass flowed from the Asian continent to the western Pacific during winter, spring and fall, whereas in summer, the wind flow pattern was from the western Pacific to the continent.

**Figure 3:**
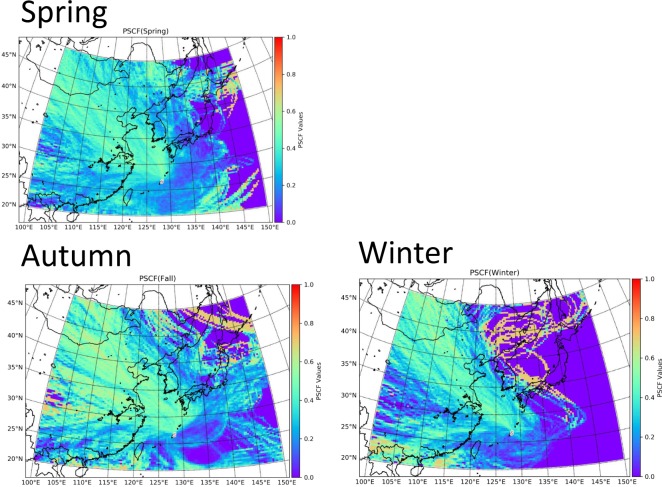
Seasonal PSCF plot of PAHs concentration over East Asia.

To understand how seasonal differences of the dominant air mass origin affected total PAH concentration at CHAAMS, we used the HYSPLIT model to calculate three-day backward trajectories for each sampling day with a starting height of 500 m (Fig. S2). The fraction of air mass origins from CH (the area including part of China) was highest in winter, whereas the fraction from the Pacific Ocean (PO) was the highest in summer (Fig. S3, Table S2). PSCF results showed that CH areas were contributing to the high PAHs concentration in spring, winter and fall (Fig. 3). Taken together, the results of Fig. 3 and Table S2 showed that the average PAH concentration was highest in winter when the air mass from CH was dominant, whereas the average concentration was lowest in summer when the air mass from PO was dominant.

**Figure 4:**
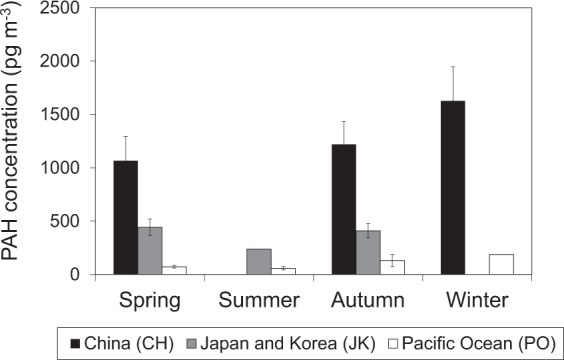
Seasonal averages of Σ15PAH concentrations of each air mass origin.

Thus, the seasonal variation of PAH concentration at CHAAMS was affected by long-range transport of PAHs from CH. The seasonal average concentration of the CH air mass was the highest followed by JK and in all seasons except for summer when no air mass was transported from CH (Fig. 4).

### Source apportionment

#### PAH compositional pattern

**Figure 5:**
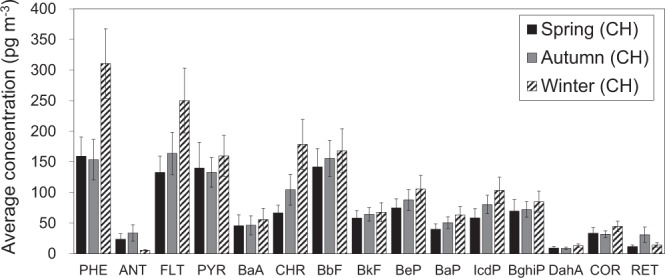
Average concentration of individual PAHs of CH origin samples in each season.

To better understand the major sources that contributed to higher PAH concentrations at CHAAMS, we focused on the datasets classified as CH in the following analyses. Detailed information including sampling period and sample number are shown in Table S3. Figure 5 shows the average concentrations of individual PAH. All of the individual PAH concentrations were higher in winter than in the other seasons, except for ANT and RET. The dominant PAHs were PHE, FLT, PYR, and CHR in winter, comprising 57% of the total PAH concentration, whereas these four PAHs made up 49% and 44% of the total concentrations in spring and autumn, respectively. Previous studies reported that PHE, FLT, PYR, and CHR are markers of coal combustion^19,20^. Other studies, however, have reported that FLT and PYR are also emitted from wood combustion and diesel engine exhaust^21,22^.

PAHs analyzed in this study can be classified according to number of aromatic rings as follows: three-ring (PHE, ANT, RET), four-ring (FLT, PYR, BaA, CHR), five-ring (BbF, BkF, BeP, BaP, DahA), six-ring (IcdP, BghiP) and seven-ring (COR). They can be further classified into lower molecular weight (LMW; three-ringed PAHs), middle molecular weight (MMW; four-ringed PAHs), and higher molecular weight (HMW; five-, six-, and seven-ringed PAHs) as discussed in Liu *et al*.^23^. In this study, the contributions of LMW + MMW PAHs were 55%, 55% and 61% of the total PAH concentrations in spring, autumn and winter, respectively. These classified rings have characteristics of PAHs’ sources. Gasoline vehicle emissions contribute significantly to four and five ringed PAHs, whereas diesel engine emissions were the dominant source of three ringed PAHs^22,24^. Recently, Li *et al*. observed PAHs in winter from 2009 to 2013 in Taiyuan City in China, where the major source was coal combustion, and reported that the fraction of LMW + MMW PAHs reached as much as 70–80% of total PAH concentration throughout the sampling period^11^. In contrast, Liu *et al*. reported that HMW PAHs were dominant and reached as much as 64–70% of the total PAH concentration in Guangzhou City, China, which is known for its heavy traffic^23^. The dominance of LMW + MMW PAHs in this study is consistent with the results of Taiyuan, especially in winter. Thus, these results suggest the dominance of coal-related PAHs transported from CH to CHAAMS, especially in winter. In addition, gas/particle partition of PAHs also contribute to the higher LMW + MMW PAHs fraction in winter than in other seasons. LMW and MMW PAHs are more volatile than HMW PAHs^25^.

#### PAH diagnostic ratio analysis

Diagnostic ratios are commonly used as an index for identifying sources of PAHs^5,26,27^. Among these diagnostic ratios, Yunker *et al*. asserted that mass 276 (e.g., IcdP, BghiP) and 202 (e.g., FLT, PYR) isomers have the greatest range in stability and hence are promising as indicators of petroleum (in other word petrogenic) versus other types of combustion sources^27^. Also, the FLT/PYR and IcdP/BghiP isomer pairs degrade photolytically at comparable rates^22^, suggesting that such ratios can be preserved during atmospheric transport. According to previous studies, FLT/(FLT + PYR) ratios < 0.40 likely imply petroleum, ratios between 0.40 and 0.50 are more characteristic of liquid fossil fuel (vehicle and crude oil) combustion, and ratios >0.50 are characteristic of grass, wood, and coal combustion^28,29^. IcdP/(IcdP + BghiP) ratios <0.20 likely imply petroleum, ratios between 0.20 and 0.50 imply liquid fossil fuel (vehicle and crude oil) combustion, and ratios >0.50 imply grass, wood, and coal combustion^30^. Since most environmental samples contain PAHs from mixed sources, we used cross plots of the FLT/(FLT + PYR) ratio and IcdP/(IcdP + BghiP) ratio for source identification^27^.

**Figure 6:**
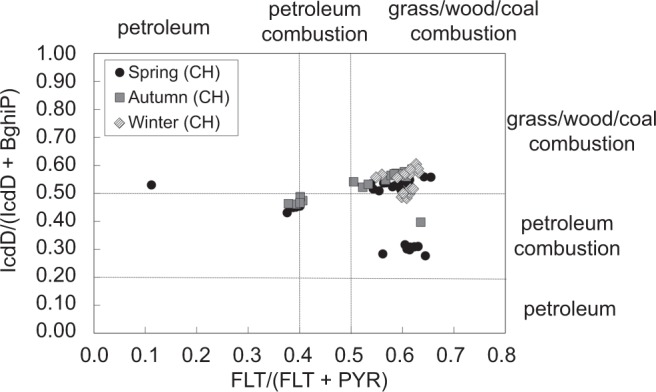
Seasonal PAH cross plots for the FLT/(FLT + PYR) and IcdP/(IcdP + BghiP) ratios for CH origin samples.

Figure 6 shows seasonal PAH cross plots for the FLT/(FLT + PYR) and IcdP/(IcdP + BghiP) ratios. The diagnostic ratios show that grass, wood, and coal combustion might be the major source in winter, whereas the sources were a mix of grass, wood, and coal combustion and petroleum combustion in spring and autumn. The winter results are in agreement with the PAH compositional pattern discussed in the previous section. Moreover, coal consumption is higher in winter, mainly due to residential heating^13^.

Figure S4 shows the variation of the two diagnostic ratios at CHAAMS. The FLT/(FLT + PYR) ratio showed no notable seasonal or annual trend for the major source and, in fact, showed a relatively constant contribution of grass, wood, or coal combustion, especially from 2009 to 2015. The IcdP/(IcdP + BghiP) ratio also implied that grass, wood, or coal combustion was a major source from 2009 to 2015. However, the ratios were lower in spring 2010 and autumn 2012, suggesting that the major source was liquid fossil fuel combustion, in contrast with the results of the FLT/(FLT + PYR) ratio for the same time periods. Similar conflicting results were also observed in Huang *et al*.^26^.

#### PMF analysis

Factor identification: The data of PAHs and trace metals (V, Ni, As, Se, Cd, Sb and Pb) in PM_2.5_ from 2010, 2012, 2013, 2014 and 2015 were used in the analysis. In particular, MMW and HMW of PAHs are mainly distributed in accumulation mode^26,31^. Thus, we consider PAHs in TSP as PAHs in PM_2.5_ and combine data of PAHs, trace metals and PAH compositional pattern. Some studies reported that PHE, FLT, PYR, and CHR are markers of coal combustion^19,20^. Other studies, however, have reported that FLT and PYR are also emitted from wood combustion and diesel engine exhaust^21,22^. Diagnostic ratios are often used to understand some specific sources of PAHs. However, the diagnostic ratios are not sufficient as conflicting results have been observed among the ratios. Therefore, combined three different source apportionment methods to identify the sources of PAHs were applied in this study. This enables us to identify major factors more clearly. We focused on data from spring and winter because in these seasons, the air mass from CH was dominant. Four factors were identified (Fig. S5).

The profile of factor 1 was dominated by PHE, ANT, FLT and RET. Factor1 was classified as a biomass burning factor. Hedberg *et al*.^21^ reported that Fluorene, PHE, ANT, FLT and PYR contribute more than 70% of the mass of PAHs emitted from wood combustion in a wood stove. Moreover, RET is known as a marker for wood combustion^32^. Moreover, previous studies which applied PMF showed similar profile for biomass burning^33^.

Factor 2 was dominated by V, Ni and ANT, and it was identified as a ship emission (fuel oil combustion) factor. V and Ni are known to be emitted from combustion of marine fuel oil. Particles emitted from ship diesel engines are also known to be mainly composed of organic carbon, sulfate and ash. V and Ni are major components of ash and are formed by burning low-grade fuel oils^34,35^. Sugiyama *et al*. identified similar profile as heavy oil combustion on ships^36^. In addition, ships using heavier residual oils as fuels are likely to have higher PAH exhaust emissions, including ANT^37^. Also, oil combustion has been reported to be associated with a high concentration of the more volatile PAHs^38,39^. In addition, the chemical reactivity of ANT in the atmosphere is the highest of the 15 PAHs detected in this study^40^. The high contribution of ANT also supports the proximity of the original emission source to CHAAMS.

Factor 3 was dominated by MMW and HMW PAHs and was classified as a vehicle emission (gasoline and diesel vehicles) factor. Among these, FLT and PYR are known as markers for diesel engine emissions^22^, whereas IcdP, BghiP and COR are typical markers for gasoline vehicle emissions^24^. Previous studies also reported that fossil fuel combustion was the main source of MMW and HMW PAHs^19,20^. Also, BbF, BaP, IcdP and DahA are typically known as markers of traffic-related emissions^20^. Chen *et al*. applied PMF for PAHs in TSP and characterized their profile with dominant IcdP and BghiP as vehicle emission factor^41^.

Factor 4 was dominated by As, Se, Cd, Sb, Pb, CHR, BbF and BkF, and was classified as a coal combustion emissions factor. Of these, As, Se, Cd and Pb are typically known as markers for coal combustion^42,43^. PHE, FLT, PYR and CHR are also known as markers of coal combustion^19,20^. In addition, CHR, BbF and BkF have also been identified as coal combustion markers^38^. Chen *et al*. identified similar profile as coal combustion factor^41^.

**Figure 7:**
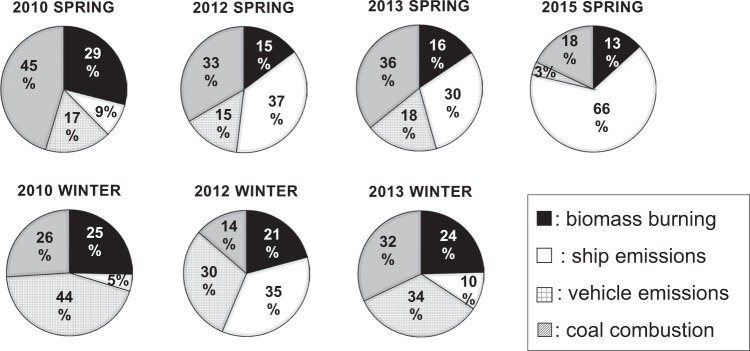
Source contributions of major sources for spring (2010, 2012, 2013, 2015) and winter (2010, 2012, 2013) from PMF model.

Seasonal changes: Figure 7 shows each source contribution in spring (2010, 2012, 2013, and 2015) and winter (2010, 2012, and 2013) evaluated by PMF analysis. In spring, biomass burning (factor 1) has contributions of 29%, 15%, 16% and 13% in 2010, 2012, 2013 and 2015, respectively. Coal combustion (factor 4) also shows a similar decreasing trend, with its contribution in 2010, 2012, 2013 and 2015 at 45%, 33%, 36% and 18%, respectively. According to the statistical data^9^, the amount of coal consumption in China gradually decreased from 2013. The total amounts of the coal consumption increased during the period, and there was no variation of the ratio of coke oven for residential area to the total coal consumption in China after 2005^44^. Thus, the decreasing tendencies were attributed to the decrease of emissions from coal combustion.

The contribution of ship emissions (factor 2) was much higher in spring than in winter; its contribution in spring of 2010, 2012, 2013 and 2015 was 9%, 37%, 30% and 66%, respectively, showing an increasing tendency from 2010 to 2015. Shipping traffic density is very high in and around the East China Sea because some of the world’s largest ports are in the coastal areas of China^45^. The amount of traffic for the ships in East China Sea has increased^46^. The number of ships in East China Sea dominates at around 30% of the total number of ships travelling^46^. Using PMF-PSCF (Potential Source Contribution Function) analysis, Shimada *et al*.^47^ show that air pollutants from oil combustion from ships sailing over the East China Sea are transported to CHAAMS. PSCF in this study also showed high contribution of PAHs concentration over the East China Sea (Fig. 3). Itahashi *et al*.^48^ also reported similar results. Wang *et al*.^49^ found a marked rise in shipping activities in the Yangtze River Delta port cluster, which has been the busiest port worldwide since 2008 as determined by estimated global ship emission inventories. Moreover, Fan *et al*.^50^ analyzed the seasonal patterns of ship emissions in the Yangtze River Delta in 2010 and concluded that the ship emissions in spring were the greatest.

In winter, the contribution of both biomass burning and coal combustion showed no notable increase or decrease trend from 2010 to 2015, contrary to the results obtained for spring. The combined contributions of these two sources were 51%, 35% and 57% in 2010, 2012 and 2013, respectively. The contribution of vehicle emissions (factor 3) was notably higher in winter (44%, 30% and 34% in 2010, 2012, and 2013, respectively) as compared with emissions in spring (17%, 15%, 18% and 18% in 2010, 2012, 2013 and 2015 respectively). According to statistical data, the possession of personal vehicles in China has been risen sharply, and the total number of vehicles had increased by about nine-fold in 2014 as compared to 2000^51^. Moreover, Ohara *et al*.^52^ projected that vehicle emissions in China in 2020 would be markedly increased, 140% (policy success case) to 220% (policy failure case) over the 1995 levels, based on a regional emission inventory in Asia. With the lack of obvious trends of contributions by biomass burning and coal combustions, these results indicate that the contribution of PAHs in winter derived from vehicle emissions at CHAAMS will most likely increase in the future.

Overall, the PMF analysis showed the high contribution of biomass burning and coal combustion both in spring and winter from 2010 to 2015, which is in agreement with the findings of the PAH compositional patterns and diagnostic ratios. The contribution of biomass burning and coal combustion together showed positive correlations with total PAH concentration, with correlation coefficients (*R*) of 0.85 and 0.98 in spring and winter, respectively. In addition, the contribution of ship emissions was high in spring and showed an increasing tendency from 2010 to 2015, whereas in winter, the contribution of vehicle emissions was high and further increases in vehicle emissions in the future are expected.

Health risk assessment: We evaluated the toxicity of PAHs by calculating the ILCR to estimate the health risk from inhalation of PAHs transported from CH. Table S4 shows the values of TEFs, estimated BaP_eq_ (pg m^−3^), and ILCR (×10^−8^) from 2008 to 2015. The highest ILCR was in 2011 (8.0 × 10^−8^). Generally, ILCR values smaller than 1 × 10^−6^ are considered as insignificant, ILCRs between 1 × 10^−4^ and 1 × 10^−6^ are considered as acceptable, and ILCRs greater than 1 × 10^−4^ are considered as significant^53^. In this study, all of the estimated ILCRs were in the insignificant range. We described the trend of ILCRs in Supplementary Information.

### Implications

Fifteen particle-associated PAH compounds in outflow from East Asia were observed at CHAAMS, Okinawa, Japan from 2008 to 2015 to evaluate the contribution of PAHs transported long-range from the Asian continent. The seasonal averages of Σ15PAH concentrations were highest in winter and lowest in summer. Backward trajectory analysis shows that the air mass from CH was dominant in winter, whereas the air mass from PO was dominant in summer. These results show that seasonal variation of PAH concentration depend largely on the air mass origin. The air mass from CH notably contributed to higher PAH concentration at CHAAMS.

In datasets of air masses classified as CH in origin, the PAH compositional pattern shows the dominance of coal-related PAHs, especially in winter. Diagnostic ratios of FLT/(FLT + PYR) and IcdP/(IcdP + BghiP) in general suggest grass, wood and coal combustion as the major source of PAHs, which is in agreement with the results of the more comprehensive PAH compositional pattern, although some conflicting ratios were observed in other seasons. PMF analysis shows the combined high contribution of biomass burning and coal combustion in both spring and winter. In addition, the contribution of shipping emissions was high in spring, whereas the contribution of vehicle emissions was high in winter.

## Methods

### Air sample collection

Particle-associated PAHs were observed at CHAAMS (128.3°E, 26.9°N), Okinawa, Japan from 2008 to 2015. Since this site is remote and has no large industrial or residential areas nearby, it is ideally located to observe PAHs transported from East Asia. Twenty-two intensive sampling campaigns were conducted from 2008 to 2015. For each sampling period, total suspended particles (TSPs) were collected on a quartz-fiber filter (QR-100, Advantec, Tokyo, Japan) using a high-volume air sampler (HV-1000F, Sibata, Tokyo, Japan) at the flow rate of 1 m^3^ min^−1^ for 24 h (in some experiments 2-day or 3-day samplings were performed; see Table S1). Before sampling, quartz-fiber filters were heated at 500 °C for 4 hours. The filter samples were separately wrapped in aluminum foil and stored in a freezer at −20 °C until analysis. Detailed information on the sampling was given in Sato *et al*. and Sugiyama *et al*.^18,36^.

### Analysis of PAHs

Procedures of extraction and analysis were nearly the same as those described in Sato *et al*. and Sugiyama *et al*.^18,36^. Briefly, each sample filter was spiked with a 100-μL isooctane solution containing a mixture of anthracene-*d*_10_, *p*-terphenyl-*d*_14_ and benz[a]anthracene-*d*_12_ (5 ppm each) as surrogates for the evaluation of PAH recovery. The filter sample was cut into 2 × 0.5 cm pieces and sonicated in dichloromethane three times (50, 60, and 70 mL) and then sonicated for 20 min in 60 mL of methanol twice to extract organic materials. Subsequently, the extracts were condensed to 3 mL by a rotary evaporator (Model R-205, Büchi Corporation, Flawil, Switzerland). Insoluble particles were removed from the concentrated extract by using a Teflon syringe filter (Biotage Isolute SPE Glass filtration 6 mL). The filtered extract was concentrated to near dryness under a gentle stream of dry nitrogen gas. The concentrated extract was then dissolved in n-hexane, and each sample was separated into five polarity fractions by using a flash chromatograph (Biotage, Isolute VacMaster-10). The first fraction of the n-hexane solution that contained PAHs was concentrated to near dryness under a gentle stream of nitrogen gas for use as the analytical sample for PAHs.

Each sample was analyzed by gas chromatography/mass spectrometry (GC-MS) (a 6890 N GC instrument combined with a 5973 Network Mass Selective Detector, Agilent Technologies, Palo Alto, CA, USA). Fifteen PAHs were detected in this study including phenanthrene (PHE), anthracene (ANT), fluoranthene (FLT), pyrene (PYR), benz[a]anthracene (BaA), chrysene (CHR), benzo[b]fluoranthene (BbF), benzo[k]fluoranthene (BkF), benzo[e]pyrene (BeP), benzo[a]pyrene (BaP), indeno[1,2,3-cd]pyrene (IcdP), dibenz[a,h]anthracene (DahA), benzo[ghi]perylene (BghiP), coronene (COR) and retene (RET). The calibration curves of the selected PAHs were measured in an injection mass region of < 1 ng by diluting a mixed authentic standard sample (Supelco, Custom Mix, 20 μg mL^−1^ each). Linear correlation coefficients between the signal intensity and the injection mass were >0.95. Lower detection limits were 5–12 pg. To check for contamination during pretreatment, a blank test was carried out for every intensive observation.

Procedural blanks were analyzed for the datasets of each sampling period. No chromatographic PAH peak was detected in the blank samples. We showed the mean recoveries of samples in Table S4.

### Air mass trajectory calculations

Three-day backward air mass trajectories from CHAAMS were calculated for each sampling day by using the HYSPLIT (Hybrid Single-Particle Lagrangian Integrated Trajectory) model (NOAA Air Resources Laboratory, Silver Spring, MD, USA; http://ready.arl.noaa.gov/HYSPLIT_traj.php). Trajectories were calculated at altitudes of 500 m above sea level. According to backward trajectories, the air mass origins were classified into three groups: China (CH), Japan and Korea (JK), and the Pacific Ocean (PO) as shown in Fig. S2. The fraction of each air mass origin was then calculated as [the number of samples from each air mass origin]/[total number of trajectories in each season].

### Pressure and winds

Figure 2 shows maps of the seasonal variation of average sea level pressure (hPa) with wind vectors (u, v) plotted using data from the National Centers for Environmental Prediction (NCEP) Final (FNL) operational global analysis data with a resolution of 1° × 1°.

### Potential source contribution function (PSCF)

We performed a PSCF analysis^54^ to identify the preferred atmospheric transport pathways from sources to receptors for PAHs concentration. PSCF estimates the conditional probability function that describes the spatial distributions of the probable source locations that contributed the pollutants observed during a given time period^54^. To investigate the transport pattern, we first calculated back trajectories such as shown in Supplementary Information (Fig. S2) but with longer residence time (5 days) and different arriving heights (500, 1000, 1500 and 2000 m above sea level) to reduce statistical uncertainty. The source domain is divided into 18000 grid cells at a resolution of 0.5° × 0.5. We described PSCF methods in Supplementary Information.

### Positive matrix factorization analysis

The Positive Matrix Factorization (PMF) model is a multivariate factor analysis tool that decomposes a matrix of speciated sample data into two matrices: factor contributions and factor profiles. The factor profiles need to be interpreted by the user to identify the source types that may contribute to the sample by using measured source profile information and emission or discharge inventories^55^.

In this study, US EPA PMF version 5.0 was used. Twenty-two columns of species were used in the PMF analysis: V, Ni, As, Se, Cd, Sb, Pb, PHE, ANT, FLT, PYR, BaA, CHR, BbF, BkF, BeP, BaP, IcdP, BghiP, DahA, COR and RET. Detailed procedures of the PMF analysis are described in Sugiyama *et al*.^36^. They used PAHs and trace metals in PMF. With the addition of trace metals in PMF modeling can identify more diverse sources compare to the PMF modeling using only PAHs. Seven trace metals (V, Ni, As, Se, Cd, Sb and Pb) in PM_2.5_ were observed at the same time as the PAHs in TSPs at CHAAMS. Since there were no trace metal analysis data in 2008, 2009, and 2011, datasets of PAHs and trace metals from 2010, 2012, 2013, 2014 and 2015 (total of 113 samples) were used for PMF analysis. The detailed sampling and analytical procedures for trace metals are described in Shimada *et al*.^47^. Aerosol particles for analyses of trace metals were collected on a Teflon filter (20.32 cm × 20.54 cm, Poreflon wp-500–50, Sumitomo Electric, Osaka, Japan) using a high-volume air sampler (Sibata, HV-1000F, Tokyo, Japan). A PM_2.5_ impactor^56^ was used in our high-volume air sampler, which was operated at a flow rate of 750 L/min. Sampling duration was 24 h. Trace metals was measured by means of inductively coupled plasma mass spectrometry (Agilent 7500 and Agilent 7700).

In this study, a four-factor solution that had the most interpretable results was selected based on Sugiyama *et al*.^46^ (Tables S6 and S7). The criteria of choosing factor number were interpretability of factors and the results of error estimation. An uninterpretable factor appeared after five factors. Four factors were the maximum number of factors which gave reasonable interpretation. We described the error estimation in Supplementary Information.

### Health risk assessment

The carcinogenic potential of BaP has been frequently studied through epidemiological and animal testing because BaP is one of the most carcinogenic PAHs. Nisbet and LaGoy developed toxic equivalency factors (*TEFs*) that compare relative toxicity of individual PAHs to BaP to assess the carcinogenic properties of each component more precisely^57^. In this study, we converted the concentrations of individual PAH into a BaP-based TEF (BaP_eq_) based on TEFs and used them as described in the literature and summarized below^11,57^. BaP_eq_ has frequently been used to indicate the potential health risk of PAHs to ecosystems and human beings^13,58^. By using the BaP_eq_ concentration, the incremental lifetime cancer risks (ILCRs) of humans were calculated. *ILCR* is the increased probability of contracting cancer over a human lifetime due to exposure to PAHs. It can be determined by calculating the lifetime average daily dose (*LADD*) of PAHs according to the US EPA guidelines^59^. The equations used to estimate *LADD* and *ILCR* are as follow:

1 $$LADD=C\times IR\times ED\times EF/(BW\times ALT)$$

2 $$ILCR=LADD\times CSF$$

where, *C* is the BaP_eq_ concentration based on *TEFs* (mg m^−3^), *IR* is the air inhalation rate (m^3^ day^−1^), *EF* is the exposure frequency (day year^−1^), ED is the lifetime exposure duration (years), BW is the body weight (kg), ALT is the average lifetime for carcinogens (days) and *CSF* is the cancer slope factor (per mg kg^−1^ day). The following values were used for calculations: *IR*, 20 (m^3^ day^−1^); *EF*, 350 (day year^−1^); *ED*, 30 (years); *BW*, 70 (kg); *ALT*, 70 × 365 (days)^59^; and *CSF*, 3.14 (per mg kg^−1^ day)^60^.

## Supplementary information

Seasonal and annual changes in PAH concentrations in a remote site in the Pacific Ocean
