# Supplementary material for: Seasonal and annual changes in PAH concentrations in a remote site in the Pacific Ocean
Source: Sci Rep. 2019 Aug 29;9:12591. doi: 10.1038/s41598-019-47409-9 (PMC6715677; doi:10.1038/s41598-019-47409-9)
Supplement: Supplementary file 1 — Seasonal and annual changes in PAH concentrations in a remote site in the Pacific Ocean [file 41598_2019_47409_MOESM1_ESM.docx]

**Supplementary Information for the manuscript:**

**Seasonal and annual changes in PAH concentrations in a remote site in the Pacific Ocean**

Kaori Miura^1^, Kojiro Shimada^2,3^, Taichi Sugiyama^4^, Kei Sato^5^, Akinori Takami^5^, Chak K. Chan^6^, In Sun Kim^7^**,** Yong Pyo Kim^2,8^, Neng-Huei Lin^2,9^, Shiro Hatakeyama^1,2,10^

*^1^Graduate School of Agriculture, Tokyo University of Agriculture and Technology, Fuchu, Tokyo, Japan*

*^2^Global Innovation Research Organization, Tokyo University of Agriculture and Technology, Fuchu, Tokyo, Japan*

*^3^Graduate School of Creative Science and Engineering, Waseda University, Tokyo, Japan*

*^4^Graduate School of Engineering, Kyoto University, Kyoto, Japan*

*^5^National Institute for Environmental Studies, Tsukuba, Ibaraki, Japan*

*^6^School of Energy and Environment, City University of Hong Kong, Hong Kong, China*

*^7^Department of Environmental Science & Engineering, Ewha Womans University, Seoul, Republic of Korea*

*^8^Department of Chemical, Engineering & Materials Science, Ewha Womans University, Seoul, Republic of Korea*

*^9^Department of Atmospheric Science and Department of Chemistry, National Central University, Chung-Li, Taiwan*

*^10^Center for Environmental Science in Saitama, Kazo, Saitama, Japan*

Correspondence and requests for materials should be addressed to K. S. (email: kshimada@aoni.waseda.jp)

Fig. S1. Seasonal averages of Σ15PAH concentrations.


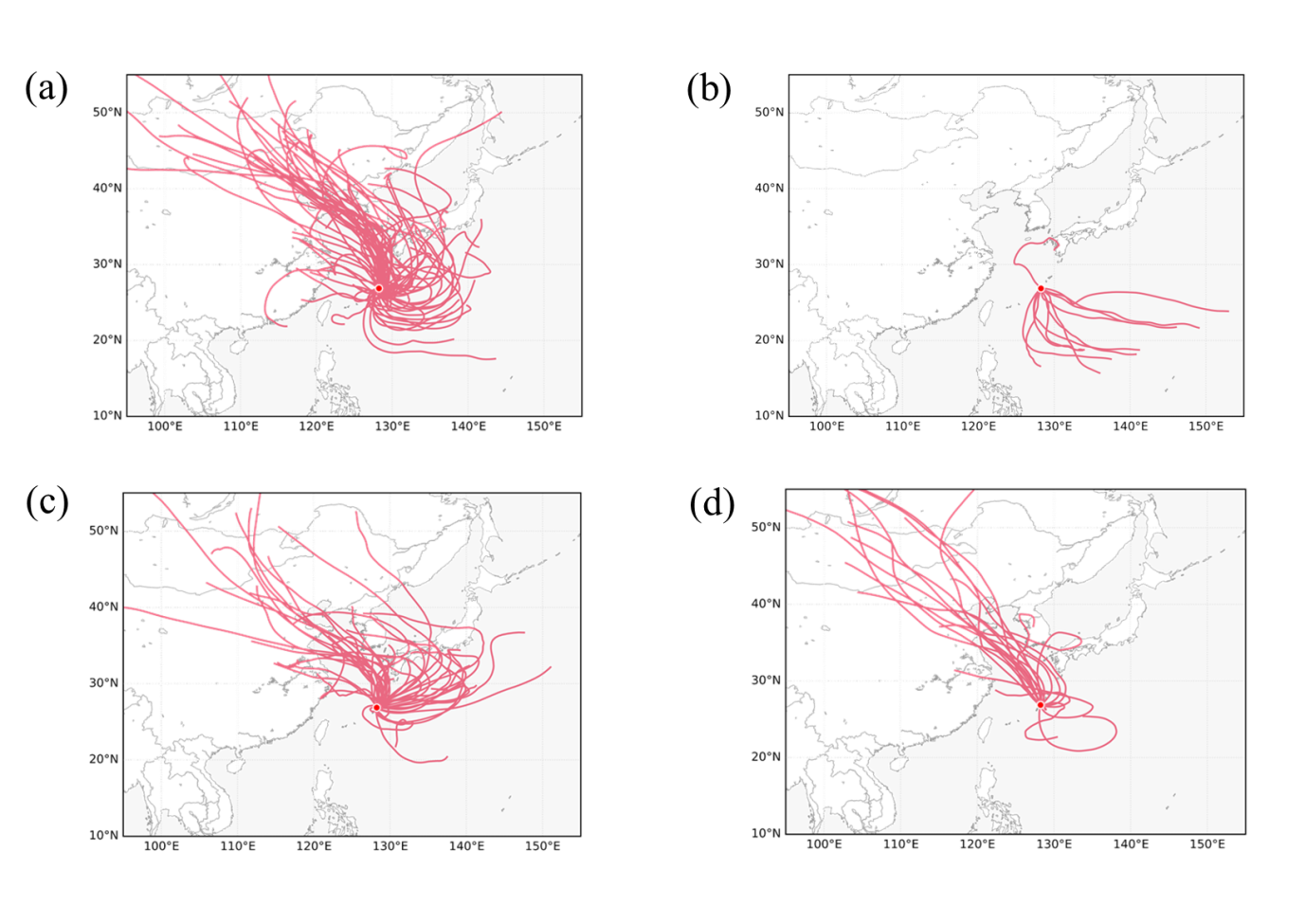


Fig. S2. Three day backward trajectories in each season; (a) spring, (b) summer, (c) autumn and (d) winter.

Fig. S3. Fraction of air mass origin by season.

 Fig. Fig. S4a. Trend of the FLT/(FLT+PYR) ratio in CH origin samples.

 Fig. S4b. Trend of the IcdP/(IcdP+BghiP) ratio in CH origin samples.


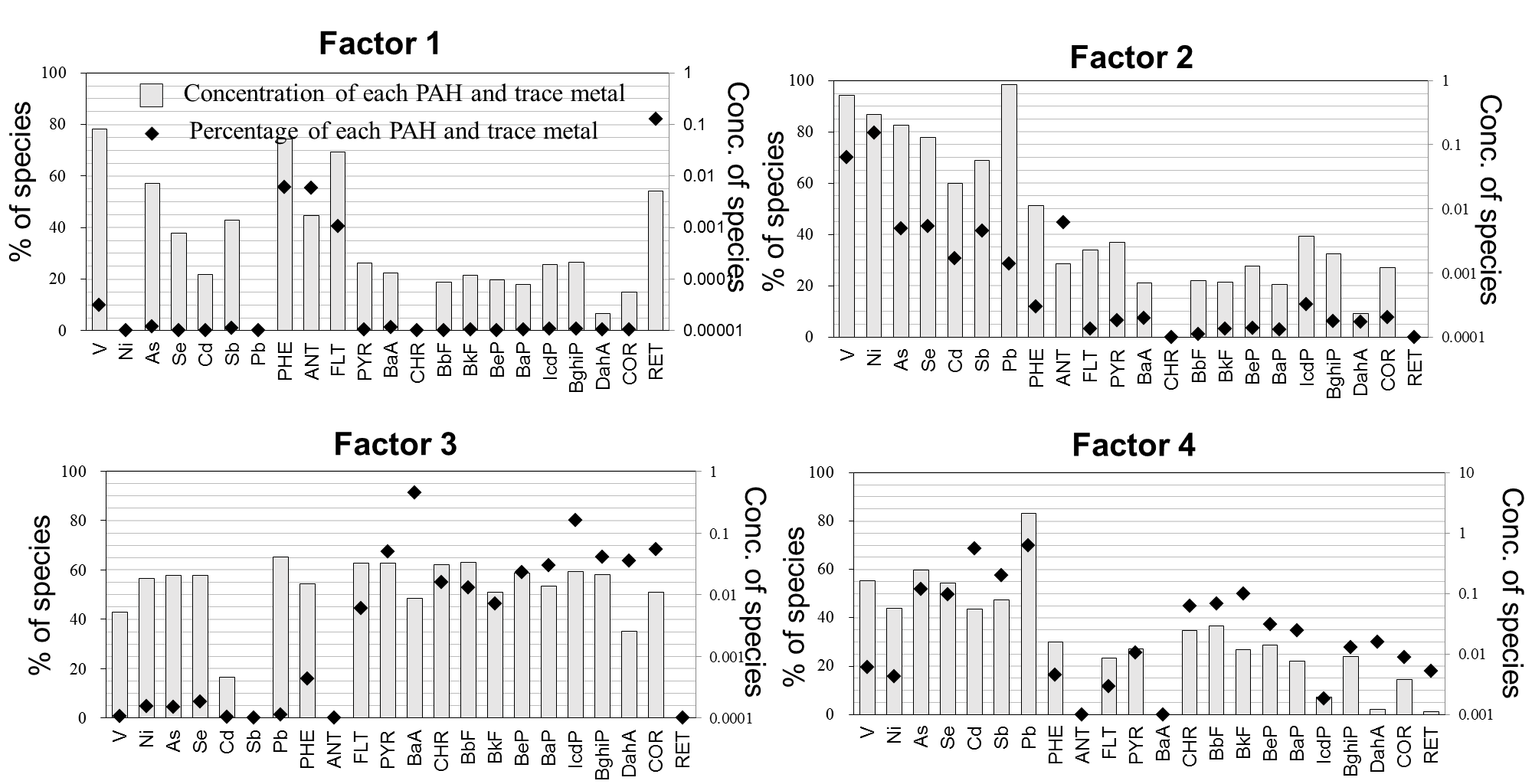


Fig. S5. Source profiles of four factors calculated by PMF; (a) factor 1: biomass burning, (b) factor 2: ship emissions, (c) factor 3: vehicle emissions, and (d) factor 4: coal combustion. Bars show the concentration of each PAH and trace metal apportioned to the factor (right axis). The squares show the percentage of each PAH and trace metal in each fraction (left axis).

Table S1. Sampling periods, sample number, TSP concentration and Σ15PAH concentration from 2008 to 2015


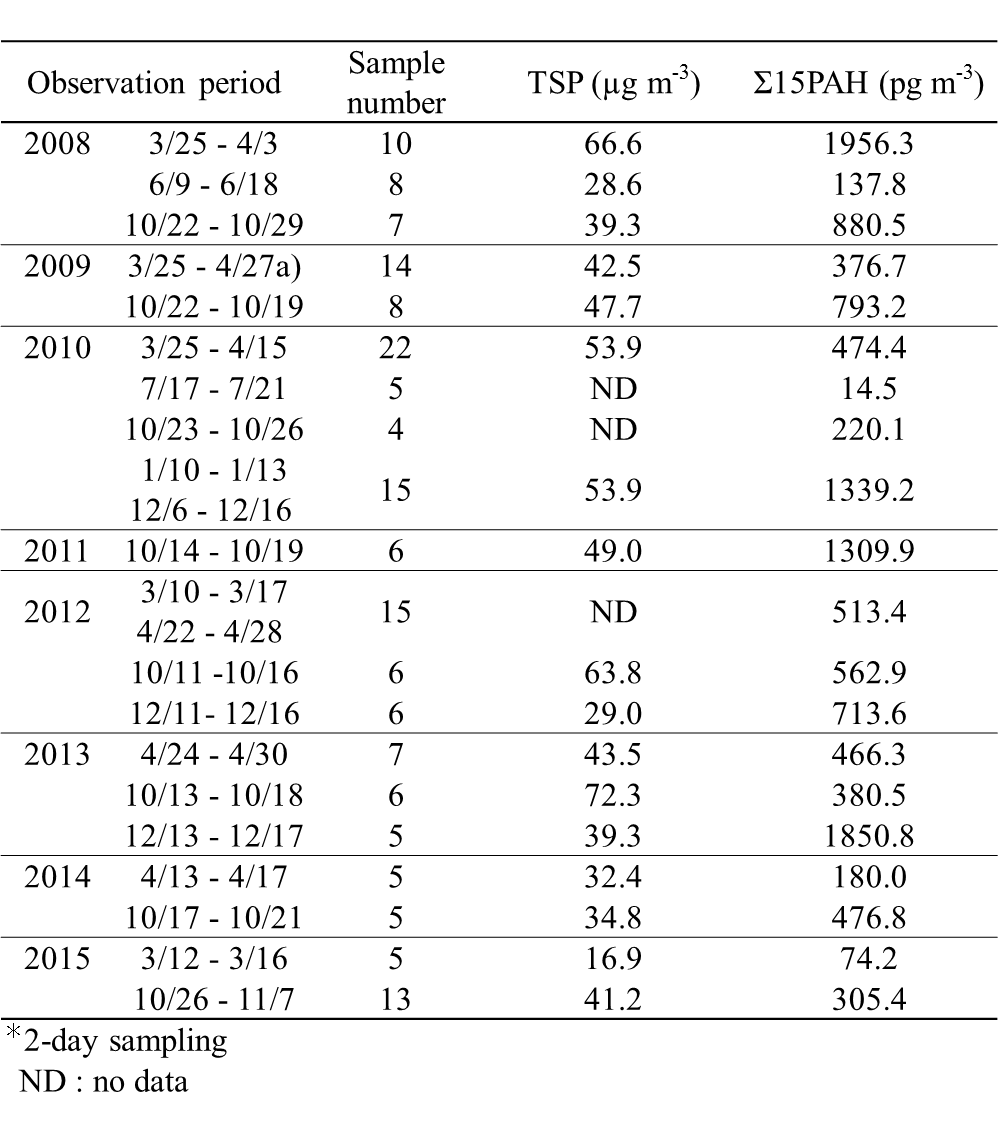


Table S2 Seasonal percentage of frequency of backward trajectories in CH, JK, PO categories Unit; Number of samples.

Table. S3 The observation period, sample number and Σ15PAH concentration between 2008 and 2015 (Focusing on CH).


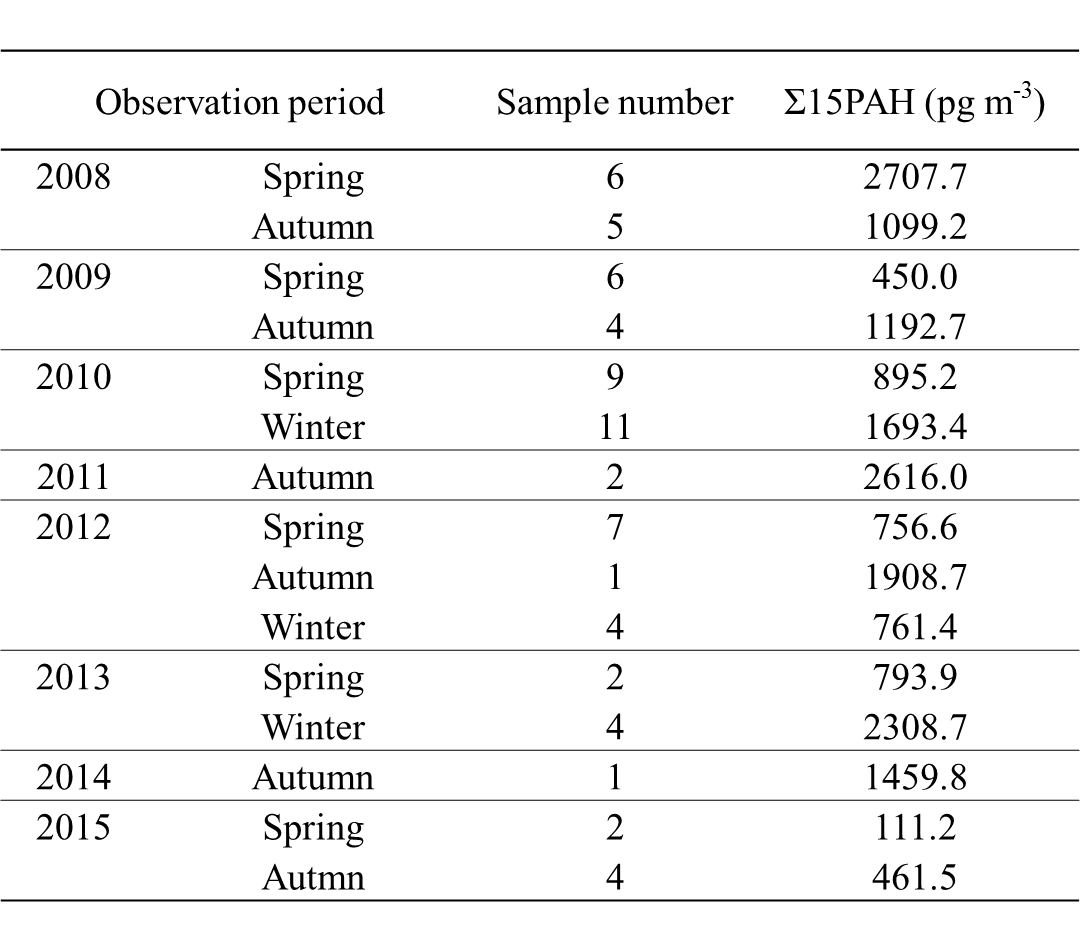


Table S4. the recovery of the surrogate standards (%).

| Surrogate standard | Mean recovery % | Range % |
| --- | --- | --- |
| anthracene-*d*_10_ | 67 | 43-83 |
| *p*-terphenyl-*d*_14_ | 107 | 73-122 |
| benz[a]anthracene-*d*_12_ | 103 | 78-115 |

**Supplementary Methods**

**Health risk assessment**

Fig. S6 and Table S5 show the annual variation of relative *ILCR* (*ILCR* of Σ12PAHs/concentration of Σ12PAHs) from 2008 to 2015. All the annual average BaP_eq_ concentrations were higher than the standard provided by European Commission (1 ng m^-3^)^1^. The relative *ILCR* values gradually increased from 2013 to 2015 despite the decreasing trend in PAH concentration. As noted previously, the amount of coal consumption has gradually decreased since 2013, but the number of vehicles has been increasing sharply since 2000^3^. Li *et al.* deduced that the increase of BaP_eq_ and *ILCR* could be related to increased vehicle exhaust because the markers of traffic emission (e.g., BbF, BaP, IcdP, and DahA) are known to be relatively more toxic PAHs (*TEF*≥0.1)^4^. Thus, the increasing trend of relative *ILCR* from 2013 to 2015 observed in this study might be due to the increased number of vehicles in China and the associated increase in emissions of toxic PAHs. If the number of vehicles in China continues to increase, the *ILCR* at CHAAMS could also rise due to an increase in transported PAHs. Therefore, in addition to PAHs related to coal combustion and biomass burning, it is also important to pay more attention to traffic-related PAHs transported from CH when considering health risks.

(a)

(b)

(c)

Fig. S6 Annual variation of (a) relative BaPeq, (b) relative *ILCR* and (c) PAH concentration.

Table. S5 The value of *TEFs* and estimated BaP_eq_ and *ILCR* (×10^-8^) concentration from 2008 to 2015

**The Method of Positive Matrix Factorization Analysis**

The Positive Matrix Factorization (PMF) model is a multivariate factor analysis tool that decomposes a matrix of speciated sample data into two matrices: factor contributions and factor profiles. The factor profiles need to be interpreted by the user to identify the source types that may contribute to the sample by using measured source profile information and emission or discharge inventories^5^.

The mathematical model in its matrix is:

$E=X-G*F$ (S1)

Where *X* is the measurement concentrations dataset matrix, *G* is the source contribution matrix, *F* is the source profile matrix, and *E* is the residual matrix.

To determine the optimal number of factors, it is necessary first to determine the minimum *Q* values for different numbers of factors. *Q* function defined as:

$$Q\left( E \right)=\sum_{i=1}^{m} \sum_{j=1}^{n} \left( E_{ij}/\sigma_{ij} \right)^{2} (S2)$$

Where *X* is the chemical component dataset matrix, *G* is the source contribution matrix, *F* is the source profile matrix, and *E* is the residual matrix.

The values $\sigma_{ij}$ are the standard deviation of the measurement concentrations value *X.* The task of the non-negatively constrained weighted factor analysis is: Minimize *Q(E)* with respect to *G* and *F* under the constraint that all or some of the elements of *G* and *F* are constrained to non-negative values. More details on PMF were described in Norris *et al* ^6^.

Different *Q* functions can be defined that *Q* (true) is the goodness of fit parameter calculated including all points. *Q* (robust) is the goodness of fit parameter calculated excluding points not fit by the model. The reduction in *Q* with the increase in the number of factors and the agreement of estimated *Q* with its theoretical value, *Q* (theory), were used to identify possible optimal solutions.

　$Q_{theory}=nm-p\left( n+m \right) (S3)$

Where *n* is number of samples, *m* is number of chemical components and *p* is number of factors^7^.

In this study, to identify the source categories through PMF analysis, we first determined 1) the optimal number of factors and 2) the stability and uncertainty of the solution. To do that, we evaluated the results of 1) base runs and 2) a bootstrap run and displacement approach (DISP). The error fraction was set to 0.15. An additional uncertainty of 13% was added to each value. For missing values, the median values of these components were used, and their errors were estimated at four times the species-specific median. If the concentration was less than or equal to the detection limit, the uncertainty was calculated as 5–6 times the detection limit^8^. Subsequently, the signal-to-noise (S/N) ratio for all data was examined to ascertain whether the measurement variability was real or within the noise level^8^; species with S/N ratios greater than 1 were considered to be “strong.” All species were set as “strong.”

To determine the optimal number of factors, various numbers of factors were tested and the resultant PMF calculation results were evaluated^9^. We performed 20 random runs and retained the runs that produced minimum *Q* values for 3–10 factors in base runs. A four-factor solution was selected based on the most physically interpretable results with the least factor smearing and most normal residuals. To estimate the stability and uncertainty in factor contributions, we performed a bootstrap model analysis and DISP. Tables S6 and S7 present the summary of the bootstrap analysis and DISP, respectively. Bootstrap analysis was performed 100 times with a minimum correlation value R = 0.6. The average percentage of bootstrap factors mapped back to the original PMF factors of this study was 91% (86%–99%; Table 2), which was higher than that of Callén *et al*. (average was 79%; 74%–90%)^10^. This indicated that our factor profile solutions were sufficiently unique. No swaps were present in all factors for dQmax 4 and 8 (Table S6). Thus, the solution was stable. These error estimations demonstrated that the model simulation results were acceptable.

Table S6 Swaps according to factors in DISP.

Table S7 Percentage of bootstrap factors mapped back to the original PMF factors from the four-factor PMF solution.

**Potential Source Contribution Function (PSCF)**

To estimate the source location of PAHs, we used the potential source contribution function (PSCF) which shows potential source areas related to high air pollutant concentrations using backward trajectories^11, 12^.

The PSCF is defined as where *n_ij_* is the number of all points in the *ij*^th^ cell; here, the number of points is usually equal to the number of back trajectories passing through the *ij*^th^ cell, but it is defined as the number of “end-points” calculated by back trajectory analyses. End-points are defined as the locations of the air mass calculated by back trajectory analyses at every hour from the start time of the trajectory. *P*[*A_ij_*]is the probability given by *n_ij_*/*N*; *N* is the total number of points summarized over all cells in the modeling region; *m_ij_* is the number of points in the *ij*^th^ cell that are classified as having a PAHs concentration exceeding the threshold value (>average PAHs concentration); and *P*(*B_ij_*) is the probability given by *m_ij_*/*N*.

$PSCF_{ij}=\frac{P[B_{ij}]}{P[A_{ij}]}=\frac{\frac{m_{ij}}{N}}{\frac{n_{ij}}{N}}=\frac{m_{ij}}{n_{ij}}$, (1)

For the PSCF calculation, the source domain was restricted to latitudes 20°N–55°N and longitudes 90°E–150°E to suppress erroneous identification of distant PSCF regions with negligible mean residence times of air masses. Backward trajectories were calculated by the HYSPLIT4 with GDAS (Global Data Assimilation System, operated by the US National Weather Service’s National Centers for Environmental Prediction) as the meteorological data. In this study, the median of PAHs concentrations was used for the threshold criterion. Five-day backward trajectories starting at every hour at a height of 500, 1000, 1500 and 2000 m above sea level were computed for every sample day, producing 120 hourly trajectory end points per sample. The geophysical region covered by the trajectories was divided into 18,000 grid cells of 0.5︒ × 0.5︒ latitude and longitude. The sources were likely to be located in the area that had high PSCF values.

To minimize the effect of small n_ij_ values resulting in high PSCF values with high uncertainties, an arbitrary weight function W(n_ij_) was applied to downweight the PSCF values for the cell in which the total number of end points was less three times the average number of end points per ^12, 13^:

$$W\left( n_{ij} \right)=\left\{ \begin{aligned} 1.0, 3\times mean(n_{ij})<n_{ij} \\ 0.7, 1.5\times mean\left( n_{ij} \right)<n_{ij}\leq3\times mean\left( n_{ij} \right) \\ 0.4, mean\left( n_{ij} \right)<n_{ij}\leq1.5\times mean(n_{ij}) \\ 0.2, n_{ij}\leq mean(n_{ij}) \end{aligned} \right.$$

PSCF was drew by a following software: anaconda package (https://www.anaconda.com/, python 2.7.14, basemap 1.0.7, matplotlib 2.1.2)

Still, the PSCF result for winter shows high probability areas north of Korean peninsula which were not shown in Fig. S2. These areas are not the real one but artificially made due to small n_ij_ values. Also, in summer, the uncertainty due to small n_ij_ values was high and made artificially high probability domains Korea and a part of China and not shown in Fig. 3.

**Supplementary References**

1. European Union (EU), 2008. Directive 2008/50/EC of the European Parliament and of the Council of 21May 2008 on Ambient Air Quality and Cleaner Air for Europe (OJ L 152, 11.6.2008). pp. 1–44 (http://ec.europa.eu/environment/air/quality/standards.htm).

2. BP. (2016). BP Statistical Review of World Energy 2016, http;//[www.bp.com](http://www.bp.com), Last Access: 21 Nov. 2016.

3. China Statistical Year Book. (2015). Department of Possession of Civil Vehicles and Total Production of Energy and Its Composition Statistics, National Bureau of Statistics, P. R. China.

4. Li, H. *et al.* A wintertime study of PM_2.5_-bound polycyclic aromatic hydrocarbons in Taiyuan during 2009–2013: Assessment of pollution control strategy in a typical basin region. *Atmos. Environ*. **140**, 404-414 (2016)..

5. Paatero, P., & Tapper, U. Positive matrix factorization: A non‐negative factor model with optimal utilization of error estimates of data values. *Environmetrics*. **5(2),** 111-126 (1994).

6. Norris, G. *et al*. EPA Positive Matrix Factorization (PMF) 5.0 fundamentals and user guide prepared for the US environmental protection agency office of research and development, Washington, DC EPA/600/R-14/108 (2014).

7. Ulbrich, I. M., *et al.* Interpretation of organic components from Positive Matrix Factorization of aerosol mass spectrometric data. *Atmos. Chem. Phys*. **9(9),** 2891-2918 (2009).

8. USEPA (2014). Positive Matrix Factorization (PMF) 5.0 Fundamentals and User Guide.

9. Bhanuprasad, S.G., Venkataraman, C. & Bhushan, M. Positive matrix factorization and trajectory modelling for source identification: A new look at Indian Ocean Experiment ship observations. *Atmos. Environ.* **42**, 4836–4852 (2008)..

10. Callén, M.S., *et al.* Nature and sources of particle associated polycyclic aromatic hydrocarbons (PAH) in the atmospheric environment of an urban area. *Environ. Pollut.* **183**, 166–174 (2013).

11. Ashbaugh, L. L., Malm, W. C., & Sadeh, W. Z. A residence time probability analysis of sulfur concentrations at Grand Canyon National Park, *Atmos. Environ.,* **19(18)**, 1263–1270 (1985)..

12. Hopke, P. K., *et al.* Possible sources and preferred pathways for biogenic and non-sea salt sulfur for the high Arctic, *J. Geophys. Res.,* **100(D8)**, 16595–16603 (1995).

13. Polissar, A. V., Hopke, P. K., & Harris, J. M. Source regions for atmospheric aerosol measured at Barrow, Alaska, *Environ. Sci. Technol.*, **35(21),** 4214–4226 (2001).
